# Correction of microplate location effects improves performance of the thrombin generation test

**DOI:** 10.1186/1477-9560-11-12

**Published:** 2013-07-05

**Authors:** Yideng Liang, Samuel A Woodle, Alexey M Shibeko, Timothy K Lee, Mikhail V Ovanesov

**Affiliations:** 1 Office of Blood Research and Review, Center for Biologics Evaluation and Research, U.S. Food and Drug Administration, 29 Lincoln Drive, N29/306, Bethesda, MD 20892, USA

**Keywords:** Thrombin generation test, Location effects, Immunoglobulin, Thrombogenicity

## Abstract

**Background:**

Microplate-based thrombin generation test (TGT) is widely used as clinical measure of global hemostatic potential and it becomes a useful tool for control of drug potency and quality by drug manufactures. However, the convenience of the microtiter plate technology can be deceiving: microplate assays are prone to location-based variability in different parts of the microtiter plate.

**Methods:**

In this report, we evaluated the well-to-well consistency of the TGT variant specifically applied to the quantitative detection of the thrombogenic substances in the immune globulin product. We also studied the utility of previously described microplate layout designs in the TGT experiment.

**Results:**

Location of the sample on the microplate (location effect) contributes to the variability of TGT measurements. Use of manual pipetting techniques and applications of the TGT to the evaluation of procoagulant enzymatic substances are especially sensitive. The effects were not sensitive to temperature or choice of microplate reader. Smallest location effects were observed with automated dispenser-based calibrated thrombogram instrument. Even for an automated instrument, the use of calibration curve resulted in up to 30% bias in thrombogenic potency assignment.

**Conclusions:**

Use of symmetrical version of the strip-plot layout was demonstrated to help to minimize location artifacts even under the worst-case conditions. Strip-plot layouts are required for quantitative thrombin-generation based bioassays used in the biotechnological field.

## Background

Thrombin generation test (TGT) measures kinetics of thrombin activity during coagulation of a blood plasma sample mixed with activators of blood coagulation [1]. The TGT is widely used in clinical research to measure global hemostatic potential in blood coagulation disorders either for diagnostic purposes [2,3] or as means of treatment monitoring [4,5]. More recently, TGT became a useful tool in drug development and control of drug potency and quality in drug manufacture [6-9]. Although proposed in the 1950s [10], the test gained popularity only a decade ago after the technique was revolutionized with the introduction of fluorogenic thrombin substrates and microtiter plate reader format [2]. However, the convenience of the microtiter plate technology can be deceiving: microtiter plate assays are prone to a special kind of variability caused by the uneven microenvironments in different wells of the plate [11].

In order to describe the location-based effects for the TGT assay, a recent biotechnology application of TGT was used. We and others found that the procoagulant activity of IVIG correlates with reported myocardial infarction, stroke and other thromboembolic events [12,13]. Commercial (CAT® by Stago and Technothrombin® by Technoclone) as well as in house variants of the fluorogenic TGT method were especially helpful in identification of procoagulant IVIG lots by manufacturers and regulatory agencies[14,15]. In this report, we describe how both in house and commercial methods are prone to location-based biases which can be addressed with the use of symmetrical strip-plot layout design.

## Materials and methods

### Materials

Human normal pooled plasma (FACT) was from George King Biomedicals. Immunodepleted Factor XI deficient plasma was from Affinity Biologicals (Ontario, Canada). Human plasma-derived Factor XIa was from Haematologic Technologies Inc (Essex Junction, VT). Recombinant lipidated tissue factor (rTF, Dade Innovin) was from Dade Behring (Marburg, Germany). TF activity was determined using the Actichrome TF chromogenic kit (American Diagnostica). Fluorogenic substrate for thrombin Z-Gly-Gly-Arg-AMC was from Bachem (Torrance, CA). Phospholipid vesicles were from Technoclone (Diapharma, West Chester, OH). The Thrombin Calibrator and TF reagent PPP-low used in the CAT instrument experiment were from Thrombinoscope BV, Maastricht, The Netherlands.

### In house thrombin generation test for IVIG procoagulant activity

Thrombin generation was measured as described in [8,16,17]. Thrombin generation profiles in FXI-deficient or normal plasma (75% by reaction volume) supplemented with fluorogenic substrate Z-Gly-Gly-Arg-AMC (800 μM) and phospholipids vesicles (4 μM) were mixed with serially diluted thrombogenic lot of IVIG or FXIa. To reduce procedural errors, all reagents and plates were kept on plate heater (37°C); plasma was mixed with substrate and lipids prior transfer to microtiter wells; IVIG and FXIa samples (20% by reaction volume) were transferred to plasma using a 12- or 8-channel pipette, and reaction was started by rapidly adding 2.54 μL (2.5% by reaction volume) of a mixture containing tissue factor (0.3 pM) and CaCl_2_ (20 mM) using another 12-channel pipette. Recording was conducted in two microplate readers, Infinite F500 (Tecan, Durham, NC) and Synergy H4 (Biotek, Winooski, VT) at 37°C.

### TG curve processing software

In house assay data processing was performed using an automated software package designed by Dr. Mikhail Ovanesov using OriginPro (OriginLab, Northampton, MA; the package is available from us upon request). The software is capable of applying different processing algorithms during the conversion of raw fluorescence to the processed TG curve, and computes the final TG curve parameters. The software can also apply the thrombin calibration and thrombin-α2macroglobulin (α2MG) correction algorithms [2]. For quantitative assessment of IVIG procoagulant activity (a bioassay approach [15]), a calibration curve was prepared from a serially diluted IVIG standard or FXIa. Thrombin peak height (TPH) values were plotted against FXIa concentration and fitted with a cubic polynomial equation. To calculate FXIa activity in the well, the calibration curve was applied to the TPH and multiplied by the pre-dilution of the sample in the well.

### Commercial thrombin generation test (CAT)

IVIG samples were mixed with plasma as described in the in house method, but after that the experiment was performed and processed according to the manufacturer’s guidelines using CAT microplate reader and software package (Diagnostica Stago, Inc., Parsippany, NJ).

## Results

### Row-dependent location artifacts of TGT

In 2010 we found that lots of IVIG products implicated in thrombotic adverse events can be evaluated with the TGT [14,15]. Mixing normal or FXI-deficient plasma with a thrombotic lot resulted in increased peak thrombin heights and shortened times to peaks [14,18]. FXI-deficient plasma provided better resolution of low and high procoagulant lots because almost no thrombin generation was observed in this plasma without added IVIG samples. To characterize the procoagulant activity of multiple IVIG products, we used thrombotic IVIG samples as internal assay controls. To simplify and standardize testing of hundreds of IVIG lots, controls were repeated on each row in the same position (i.e., as “row controls”), as shown in Figure 1A. This arrangement of samples is consistent with the high-throughput implementation of the manual TGT that utilizes 12-channel pipette for quick recalcification of samples in one row [8]. Consistently with the use of the row-to-row recalcification strategy, we found some random row-to-row variability in TPHs between and within microplates (data not shown). These differences could be traced back to inaccurate dispensing of TF and calcium chloride reagent.

**Figure 1:**
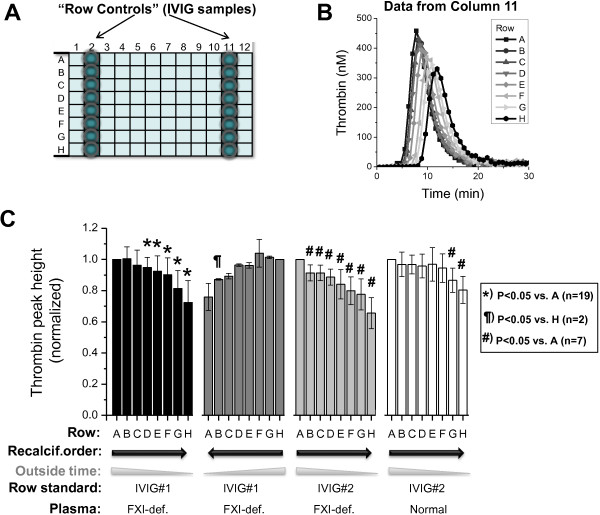
**Row artifacts (location effects) in the TGT assay for procoagulant activity of immune globulin products. *A***. 96 well microplate diagram showing location of row standards (shaded circles) included in each TGT experiment. ***B***. Effect of row location on the thrombin generation curve in a typical experiment. ***C***. Effect of row location on thrombin peak height (TPH) under a variety of conditions. TPHs were normalized on the first recalcified row (either row A or H). Means ± S.D. are shown. *Black bars*: *n* = 19 assays; FXI-deficient plasma (FXIDP) mixed with IVIG sample #1. Order of recalcification: row A ➔ row H. *Dark gray bars*: *n* = 2; conditions as in black bars but the order of row recalcification is reversed (row H ➔ row A). *Light gray bars*: *n* = 7; conditions as in black bars but IVIG sample #2 (higher activity) was used. *White bars*: *n* = 7; conditions as in light gray bars but normal pooled plasma was used. Symbols above bars indicate statistically significant difference versus first recalcified row using paired *t*-test (P < 0.05).

However, we found that TG in duplicate wells containing the same IVIG sample can differ in a non-random systematical manner by up to 50% if the wells are located on separate rows of the plate. This can be illustrated by progressively reduced and delayed TG of “row control” samples on rows A through H (Figure 1B). Since lag time and time to thrombin peak were affected greater than the TPH by this systematic error, we chose TPH as the primary measure of IVIG procoagulant activity.

Analysis of multiple TGT microplate experiments presented in Figure 1C demonstrates statistically significant trends between upper and lower rows of the microplate under variety of conditions. Similar trends were observed for two IVIG samples (Figure 1C, compare black and light gray bars). Normal plasma (white bars) was less sensitive to this trend which correlates well with lower sensitivity of normal plasma to procoagulant activity under the conditions of this experiment (data not shown). Interestingly, when the order of row recalcification was reversed from A ➔ H to H ➔ A, the trend was also reversed (Figure 1C, compare black and dark gray bars).

### Effect of temperature

Previous investigations demonstrated that reduced temperature of plasma sample prior to placement of the microplate into the reader may lead to increased thrombin generation [19]. However, the role of temperature in the observed row-to-row artifacts was ruled out in our experiments. We kept our plasma and microplates on heat plates (three different brands of heaters used) and used pre-warmed pipet tips and microplate readers. The temperature of plasma wells on the heater before, after recalcification and after readings in the microplate reader was the same as assessed by an infra-red thermometer (0.1°C resolution).

### Row effect on IVIG samples with different procoagulant activities

Normalization of the sample responses on the response of internal standard control can be used to address random and systematic errors in biological assays. However, normalization of TG peak heights in various tested IVIG samples on the respective “row control” presented in Figure 1 failed to correct for row-to-row artifacts (data not shown). Further analysis revealed that normalization is ineffective because samples with high and low procoagulant activities are affected differently by row artifact. To model samples of different procoagulant activity, procoagulant IVIG lot was serially diluted and tested repeatedly on rows A through H (Figure 2A). On each row, the TG curves demonstrated clear dose-response (Figure 2B). Consistent with previous observations, there was significant row-to-row random error (e.g., row C lower than row D on Figure 2C) and a systematic trend across different rows (reduction of TPH from row A to row H). More importantly, highly concentrated IVIG sample was less affected by the systematic row drift than highly diluted samples, as evidenced by different slopes of respective TPHs (compare blue and red lines on Figure 2D). Therefore, a procoagulant activity calibration curve rather than normalization on a single concentration of row control should be used for correction of row artifacts.

**Figure 2:**
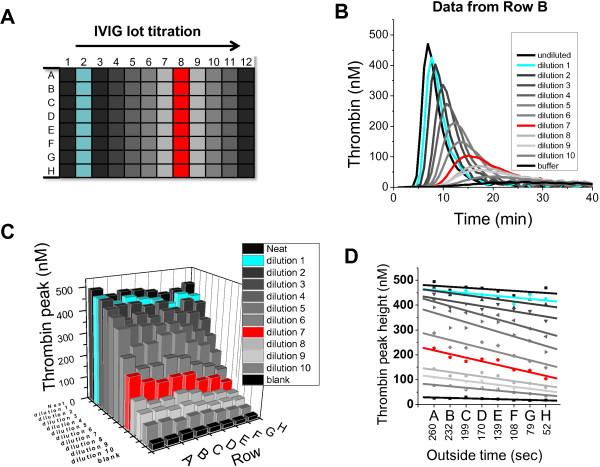
**Random and systemic location effects observed for serially diluted procoagulant IVIG product. *A***. 96 well microplate diagram showing row-to-row location of serially diluted IVIG sample. Blue and red colors on this and other panels denote 1.6- and 16.8-fold dilutions, respectively. ***B***. Thrombin generation profiles in FXIDP mixed with serially diluted thrombogenic lot of IVIG. Data from row **B**. ***C***. Effect of row location on the TPH measured in the TGT assay. Note random row variations, e.g., relatively low thrombin peaks on rows B and F, and systemic trend to TPH reduction on rows **A** ➔ **B**. ***D***. Linear fit was used to demonstrate that row-to-row drift is dependent on the activity of the sample and time between row recalcification and placement into the reader (outside time).

### Row effects for FXIa on different microplate reader instruments

Microplate reader models differ in the patterns of microplate movement, vibration rate and flow of heated air inside the instrument, possibly changing the kinetics of coagulation in microwells, e.g., “edge” artifacts of variable intensity. However, we found that two different microplate readers produced similar row-to-row artifacts when the same set of samples was tested in parallel (Figure 3C). Note that these experiments utilized titration of FXIa, a coagulation protein previously identified as the primary thrombogenic substance in IVIG products [14,15,18]. Procoagulant IVIG lot and purified FXIa produced similar dose responses in the TGT (compare Figures 2B and 3B) and similar row-to-row drifts (Figures 2D and 3C).

**Figure 3:**
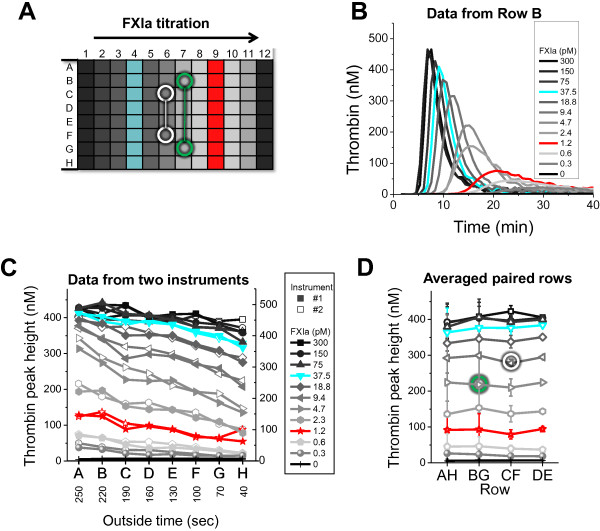
**Similar row artifacts observed on two different microplate reader instruments. *A***. 96 well microplate diagram showing row-to-row location of FXIa. Blue and red colors on this and other panels denote 37 and 1.2 pM of FXIa (sample concentration before it was added to plasma). ***B***. Thrombin generation profiles in FXIDP. Data from row **B**. ***C***. Effect of row location on the TPH measured in two parallel TGT assays on two microplate reader instruments. ***D***. Averaging data from symmetrical rows A/H, B/G, C/F and D/E corrects the row trend artifact. Means ± S.D. (*n* = 2 wells).

### Correction of row-to-row artifacts with symmetrical duplicate well averaging

Systematic analysis of analytical and preanalytical variables revealed that the time period from recalcification of the well to placement of the microtiter plate into the plate reader (outside time) correlates with the TPH (e.g., see Figures 1C, 2D and 3C). Therefore, averaging identical samples on opposite rows, e.g., rows C + F and rows B + G (see white and green connected circles on Figure 3A diagram), compensated for the row drift at all FXIa concentrations (Figure 3D). In practice, quantitative assessment of procoagulant samples against the activity control would require serial dilutions of samples and controls to be arranged vertically and symmetrically with respect to the center of the microplate, as shown on Figure 4A diagram. To confirm utility of this approach, the IVIG sample dilutions from the experiment shown on Figure 2 were retested using the symmetrical vertical strip plot design (Figure 4A), resulting in correction of row artifacts (Figure 4B).

**Figure 4:**
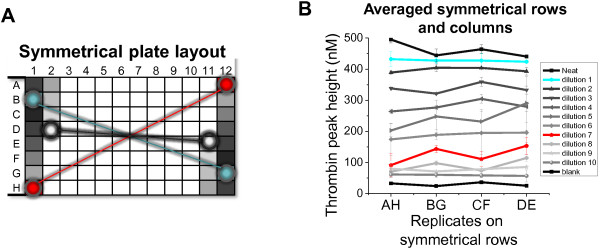
**Symmetrical strip**-**plot layout for duplicate wells corrects for row artifacts. *A***. Symmetrical plate layout design in which duplicate wells are positioned symmetrically with respect to the plate center. Blue and red colors on this and other panels denote 1.6- and 16.8-fold dilutions, respectively. Colored circles denote three examples of duplicate wells: B1 and G12, H1 and A12, and D2 and E11. ***B***. Correction of row-to-row location effects in samples arranged symmetrically. The conditions of the experiment are similar to shown on Figure 2 with the exception of a different layout design. Means ± S.D. (*n* = 2 wells).

### Row effects in a commercial TGT

Since row drifts were proportional to recalcification times, fast recalcification may improve row artifacts. Consistent with this prediction, we found less pronounced location-dependent effects in a commercially available CAT® variant of the TGT assay which is equipped with the automated calcium chloride dispenser. Separate experiments confirmed that other differences between in house and CAT assays (e.g., order of substrate addition, volume of plasma, TF concentration) could not explain the different degree of row effects (data not shown). To understand the difference between adjacent and symmetrical averaging of duplicate wells, we placed identical FXIa titrations along every column and the direction of the titration along the column was symmetrical with respect to the center of the plate (refer to Figure 5A). This order is roughly consistent with the left-to-right direction of column-wise recalcification order by the automated CAT instrument. Blue lines on Figure 5B demonstrate column drifts when adjacent duplicate wells containing same FXIa concentrations are averaged (examples of adjacent wells are marked with blue circles on Figure 5A). The drifts were eliminated when symmetrically arranged wells were averaged (Figure 5A, orange circles, and Figure 5B, orange lines). Furthermore, symmetrical averaging of wells produced overlapping dose-response curves for FXIa-dependent TPH (Figure 5D), while adjacent averaging produced shifted dose-response curves (Figure 5C).

**Figure 5:**
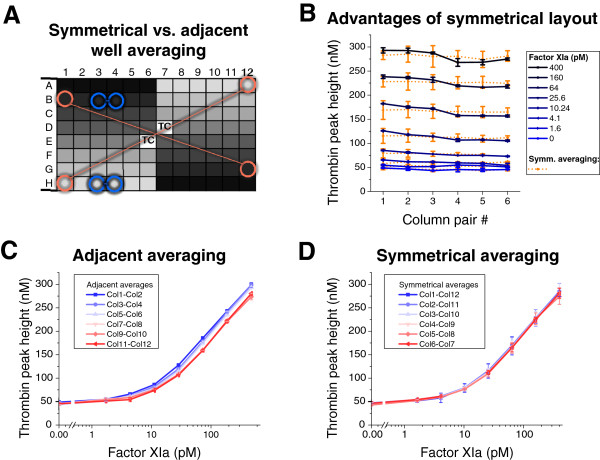
**Location effects in the commercial thrombin generation assay (CAT). *A***. Symmetrical plate layout design in which FXIa titration was repeated on columns 1 through 6, and, in reversed direction of sample dilutions, on columns 7 through 12. Colored circles denote two algorithms of duplicate well averaging: *orange circles* demonstrate symmetrically positioned duplicate wells; *blue circles* demonstrated adjacent position of duplicate wells. “TC” denotes thrombin calibrator wells. ***B***. Adjacent averaging (blue lines) reveals column drifts in TPH while symmetrical averaging of duplicate samples corrects this trend (orange lines). Means ± S.D. (*n* = 2 wells). ***C***. Effect of column location on the FXIa dose - TPH response curves in the case of adjacent averaging. ***D***. Symmetrically averaged columns overlap indicating corrected location artifacts. Means ± S.D. (*n* = 2 wells).

### Importance of row effect correction for quantitative bioassay

To estimate the potential bias in a FXIa bioassay [15] produced by the row and column artifacts, we utilized the data from the CAT experiment (Figure 5) that demonstrated the lowest systematic drift of all experiments. The data from the first column or averaged data from symmetrical columns 1 and 12 was used as a calibration curve, as shown on Figure 6A and B. Although the difference between fitted curves was seemingly small, the activity of FXIa dilutions on columns from left to right experienced progressive reduction up to 30% (Figure 6C, black line). In contrast, symmetrical averaging eliminated this trend completely (gray line).

**Figure 6:**
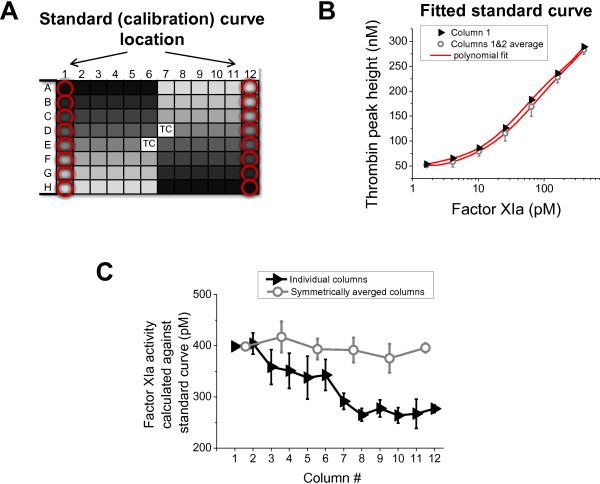
**Biases introduced by location artifacts in the commercial CAT instrument. *A***. Plate layout demonstrating two locations of calibrations curves, column 1 vs. averaged symmetrical columns 1 + 12, used for bioassay analysis in this figure. “TC” denotes thrombin calibrator wells. ***B***. Calibration curves from column 1 (filled symbols) and averaged columns 1 + 12 (open symbols) fitted using polynomial equations (red lines). Means ± S.D. (*n* = 2 wells). ***C***. Calculated FXIa concentrations obtained for either individual columns 1 through 12 against calibration curve from column 1 (filled symbols) or for averaged symmetrical columns 1 + 12, 2 + 11, etc. against the calibration curve from columns 1 + 12. The activities of FXIa samples were calculated under the assumption that each column contained a single sample that was serially diluted, starting with the highest “neat” sample in the first well of the respective column, followed with 2.5 dilutions for wells 2 through 7 (well 8 was “blank” buffer control). In the bioassay, TPH of each well in the column series was compared with the calibration curve (Figure 6B), and resultant FXIa concentration was multiplied by the respective dilution factor. Thereafter, the FXIa data from the first 6 wells were averaged, and S.D. was calculated. The highest diluted sample in well 7 was ignored because it was too close to the limit of assay detection.

## Discussion

The clinical laboratory version of the TGT is usually intended for comparison of coagulation potentials of patient and healthy donor plasma samples [2,3] or patient-specific evaluation of the procoagulant and anti-coagulant effects of existing and investigational treatments [4,5]. Consequently, prior research on TGT assay development largely focused on preanalytical conditions and proper calibration of assay in units of thrombin activity. Improved quality of collected blood plasma samples, control of plasma temperature as well as internal thrombin calibration or normalization on standard plasma sample were shown to improve assay performance.

Evaluation of drug efficacy and quality, unlike clinical applications, may be less sensitive to preanalytical conditions because several drug preparations are compared with each other on the same microplate using a single sample of plasma. However, quantitative assessment of the difference between drug preparations usually requires comparison of different drug doses. Ideally, a bioassay is employed in which comparison to a standard drug preparation employs a calibration curve or a parallel line assay. Therefore, biotechnology applications require additional TGT qualification to ensure consistent low limit of detection, linear dose-response range and parallelism of tested and standard drugs.

Randomization of samples is an effective solution to correct location effects in high throughput analytical assays, but complete randomization of sample layout on the microtiter plate is prone to procedural errors [20]. A more practical approach is a strip-plot design in which samples are assigned to random columns and serial dilutions of each sample to one column [20]. However, in a typical TGT experiment, more samples are tested than there are columns on a plate, and the number of serial dilutions is smaller than there are rows. Since all coagulation samples are tested at least in duplicates, we propose to use a symmetrical design in which duplicates are positioned symmetrically with respect to the center of the plate. When applied to IVIG-TGT, this approach corrects the row-to-row drift and is easy to implement: (a) prior to addition to plasma, double volumes of IVIG samples are arranged in columns 1 through 6 (half of the plate), each sample in a single well, dilutions arranged vertically, (b) plasma is mixed with other required reagents (e.g., lipids) and transferred to cover all 96 wells of another plate, (c) IVIG samples are transferred to columns 1 through 6 of the plasma plate, (d) the plasma plate is then rotated 180° degrees, and (e) step *c* is repeated.

While we encountered location errors during drug screening studies, these findings may be even more important for other TGT applications, e.g., use of TGT for diagnosis and treatment of patients [5]. Sensitivity of coagulation assays to pre-analytical conditions has been known for decades yet the causes of TGT variability have only recently been investigated [19], but location artifacts were not discussed in the literature. Location effect can be introduced in various ways. We found that if a coagulation enzyme, e.g., FXIa or FVIIa, is added manually to multiple wells on a microtiter plate, a well-to-well drift appears due to the different durations of contact between the enzyme and plasma inhibitors. A similar effect may be observed if TF or contact activator is added manually before recalicification. In our experience, long exposure of plasma to fluorogenic substrate prior to recalcification also decreases the TGT response. Edge effects can be caused by uneven heating of microplate inside the reader and artifacts of liquid dispenser. It should be noted that internal normalization on the thrombin-α2macroglobulin activity calibrator (e.g., as proposed by Hemker et al. [21]) is not helpful here because the thrombin calibrator only corrects for fluorogenic signal-related inaccuracies and fails to correct for biological and pipetting variables [22]. All location effects may be corrected with the use of the strip-plot sample design. Specifically, when edge artifacts are minor, our symmetrical strip-plot design provides the simplest practical solution for microplate-based TGT experiments.

## Competing interests

The authors declare that they have no competing interests.

## Authors’ contributions

Dr. MVO designed the study, developed thrombin generation analysis software and wrote the paper. Dr. YL and SAW conducted the experiments presented in this paper. Dr. AMS conducted preliminary experiments and assisted with method development. Dr. TKL contributed to the study interpretations and helped writing the paper. All author’s read and approved the final manuscript.
